# Simultaneously Determined Antioxidant and Pro-Oxidant Activity of Randomly Selected Plant Secondary Metabolites and Plant Extracts

**DOI:** 10.3390/molecules28196890

**Published:** 2023-09-30

**Authors:** Tibor Maliar, Mária Maliarová, Marcela Blažková, Marek Kunštek, Ľubica Uváčková, Jana Viskupičová, Andrea Purdešová, Patrik Beňovič

**Affiliations:** 1 Department of Chemistry and Environmnetal Sciences, Faculty of Natural Sciences, University of Ss. Cyril and Methodius in Trnava, Nám. J. Herdu 2, 917 01 Trnava, Slovakia; maria.maliarova@ucm.sk (M.M.); andrea.purdesova@ucm.sk (A.P.); benovic2@ucm.sk (P.B.); 2 National Agricultural and Food Centre, Hlohovecká 2, 951 41 Lužianky, Slovakia; marcela.blazkova@nppc.sk (M.B.); marek.kunstek@nppc.sk (M.K.); 3 Department of Biology and Biotechnology, Faculty of Natural Sciences, University of Ss. Cyril and Methodius in Trnava, Nám. J. Herdu 2, 917 01 Trnava, Slovakia; lubica.uvackova@ucm.sk; 4 Centre of Experimental Medicine SAS, Institute of Experimental Pharmacology and Toxicology, Slovak Academy of Sciences, Dúbravská cesta 9, 841 04 Bratislava, Slovakia; janaviskupicova@gmail.com

**Keywords:** oxidative stress, antioxidant activity, pro-oxidant activity, compounds, plant extract

## Abstract

Oxidative stress is a well-known phenomenon arising from physiological and nonphysiological factors, defined by the balance between antioxidants and pro-oxidants. While the presence and uptake of antioxidants are crucial, the pro-oxidant effects have not received sufficient research attention. Several methods for assessing pro-oxidant activity, utilizing various mechanisms, have been published. In this paper, we introduce a methodology for the simultaneous determination of antioxidant and pro-oxidant activity on a single microplate in situ, assuming that the FRAP method can measure both antioxidant and pro-oxidant activity due to the generation of pro-oxidant Fe^2+^ ions in the Fenton reaction. Systematic research using this rapid screening method may help to distinguish between compounds with dominant antioxidant efficacy and those with dominant pro-oxidant effects. Our preliminary study has revealed a dominant pro-oxidant effect for compounds with a higher number of oxygen heteroatoms, especially sp2 hybridized compounds (such as those containing keto groups), such as flavonoids and plant extracts rich in these structural types. Conversely, catechins, carotenoids, and surprisingly, extracts from birch leaves and chestnut leaves have demonstrated dominant antioxidant activity over pro-oxidant. These initial findings have sparked significant interest in the systematic evaluation of a more extensive collection of compounds and plant extracts using the developed method.

## 1. Introduction

The commercial database SciFinder, one of the most widely utilized databases, reveals a significant lack of scientific papers on pro-oxidants. Keyword searches for “ANTIOXIDANT/PROOXIDANT/ANTIOXIDANTS and PROOXIDANTS” in titles yielded 55,177 records for antioxidants, 542 records for pro-oxidants, and only 227 records for papers mentioning both antioxidants and pro-oxidants. The database categorizes these results into manuscripts, patents, reviews, clinical studies, books, conference contributions, etc. A similar situation arises when searching for antioxidant/pro-oxidant activity in plant extracts. To provide a systematic overview of this field, it is essential to describe and explain the FRAP method applied in this paper. FRAP stands for “Ferric Reducing Antioxidant Power”. This assay measures antioxidant power by reducing ferric-tripyridyltriazine (Fe^3+^-TPTZ) to an intense blue-colored ferrous-tripyridyltriazine complex (Fe^2+^-TPTZ) at a low pH, with an absorption maximum of 593 nm [1]. Trolox is commonly used as a positive control, and results can be expressed as μM of Trolox equivalents or μM Fe^2+^ based on a standard curve. The FRAP assay is a proven method for assessing the antioxidant capacity of foods and legume seeds, which is closely related to their polyphenol contents [1]. In 1996, Benzie and Strain developed the FRAP assay to estimate the ferric-reducing power of human plasma [2]. Dragsted et al. [3] adapted the FRAP assay for use with a microtiter plate reader in a 96-well format. Their team successfully determined the FRAP values of plant extracts and agrarian crop extracts using a slightly modified method on microtiter plates [4,5,6,7,8]. In the paper by Wojtunik-Kulesza [9], it was highlighted that the FRAP assay requires specific conditions, including an acidic medium (pH 3.6) to facilitate iron solubility and a temperature of 37 °C. The low pH reduces the ionization potential, promoting electron transfer and increasing the redox potential, which affects the dominant reaction mechanism [10,11]. However, it is essential to note that the FRAP assay, similar to other assays, has its limitations. The redox potential of the Fe^3+^/Fe^2+^ pair plays a crucial role, as compounds with a lower redox potential may yield falsely high Fe^3+^ reduction results. Additionally, FRAP assay results depend on the timescale of the analysis [11].

In the field of antioxidant and pro-oxidant activity methods, numerous papers and concepts have been introduced. In brief, reduction of a chemical refers to a gain of electrons, while oxidation refers to a loss of electrons [12]. A reductant or reducing agent donates electrons, causing another reactant to be reduced, while an oxidant or oxidizing agent accepts electrons, leading to the oxidation of another reactant. These terms have specific meanings in the context of a biological system [12]. Based on this perspective, assays can be divided into two categories: antioxidant capacity assays involving oxidants that are not necessarily pro-oxidants, and antioxidant capacity assays involving oxidants that are pro-oxidants, as published in [1,13]. The FRAP assay falls under the first category, as it involves an oxidant, Fe^3+^. However, it is essential to note that Fe^3+^ is not necessarily a pro-oxidant. On the other hand, Fe^2+^, produced from the reduction in Fe^3+^ in the FRAP assay, could act as a pro-oxidant due to its reaction with H_2_O_2_. Nevertheless, neither Fe^2+^ nor Fe^3+^ directly cause oxidative damage to lipids, proteins, or nucleic acids.

The explanation of Fenton reactions and the significance of Fe^2+^ and Fe^3+^ ions in these reactions is crucial for understanding the subsequent discussions. The Fenton system uses ferrous ions (Fe^2+^) to react with hydrogen peroxide (H_2_O_2_), producing hydroxyl radicals (OH^●^) and hydroxide ions (OH^−^) (Equation (1)). Fenton-like reactions involve a two-step system, generating hydroperoxyl radicals (HO_2_^●^) in the subsequent reactions (Equations (2) and (3)).

H_2_O_2_ + Fe^2+^ → Fe^3+^ + OH^●^ + OH^–^ (1)

The hydroxyl radical is a very reactive oxidant capable of rapidly reacting with surrounding molecules.

H_2_O_2_ + Fe^3+^ → Fe···OOH_2_^+^ + H^+^ (2)

Fe···OOH^2+^ → Fe^2+^ + HO_2_^●^ (3)

The significance of Fenton-like reactions lies not only in the production of the HO_2_^●^ radical but also in the conversion of ferric ions (Fe^3+^) to ferrous ions (Fe^2+^), which can initiate another Fenton reaction cycle. The kinetics of Fenton oxidation are complex and can be described by a combined pseudo-first-order kinetic model, while Fenton-like reactions follow simpler, pseudo-first-order kinetics [14]. However, several studies have shown that the rate of decomposition of H_2_O_2_ and the rate of oxidation of organic solutes are much slower using Fe^3+^/H_2_O_2_ than Fe^2+^/H_2_O_2_ as a source of radicals [15]. In Fenton-like reactions, ferric ions react with H_2_O_2_ to produce ferrous ions at a very slow rate (k = 0.001–0.01 M^−1^ s^−1^) [16]. Macáková et al. [17] published a paper describing that iron reduction potentiates hydroxyl radical formation only in flavonols. Flavonoids, substantial components of the human diet, are generally considered beneficial. However, they may possess possible pro-oxidative effects based on their reducing potential. The study revealed that a substantial reduction of ferric ions occurred under acidic conditions, particularly with flavonols and flavanols containing the catecholic ring B. This paper, along with others, sheds light on the interactions between flavonoids and iron, bringing another dimension to the understanding of these reactions. The findings showed that flavonols such as morin and rutin exhibited progressive pro-oxidant effects, while 7-hydroxyflavone and hesperetin were the only flavonoids with dose-dependent inhibition of hydroxyl radical production [17].

As far as the authors are aware, there has not been a paper published describing the FRAP assay as a method for the simultaneous determination of both antioxidant and pro-oxidant activities of tested samples based on the conversion of Fe^3+^ to Fe^2+^. The ferrous ion is currently recognized as the more pro-oxidative form of iron. However, there are similar methods based on the same principle, involving the colorization of some transition metals. Many of these methods employ both the ferrous and ferric ions in the complex for spectrophotometric determination and analysis. Particularly, several methods for determining pro-oxidant activity have been published, such as the method of reducing power (RP) using potassium ferricyanide [18], the Ferric-ferrozine assay for total antioxidant capacity using ferrozine as a ligand [19], the CUPRAC method using neocuproine as a ligand [20], or the copper reducing activity index (CRAI) assay using sodium diethyldithiocarbamate as a ligand (DDTC) coupled with TBARS determination of pro-oxidative fragments of linoleic acid [21].

In addition to the methods mentioned earlier, several techniques are available for quantifying the pro-oxidant effect on DNA, proteins, or lipids [22], or determining the pro-oxidant effects on cell morphology in vitro [23,24].

The simultaneous detection of both antioxidant and pro-oxidant activities has been published. Some papers used peroxidase (myeloglobin/H_2_O_2_)-generated ABTS^+●^ [2,2′-azinobis-(3-ethylbenzthiazoline-6-sulfonic acid)] radical cation [25], the β-carotene bleaching assay [25], and the crocin bleaching assay [26]. In this paper, we present a simultaneous determination of antioxidant activity using the DPPH method and antioxidant pro-oxidant activity of compounds and extract samples using the FRAP method, modified on a microplate.

Antioxidative active compounds and well-known antioxidants, including vitamins, may also act as pro-oxidants, depending on their concentration. For instance, vitamin C is a potent antioxidant, but it can exhibit pro-oxidant behavior depending on the dosage [27]. The pro-oxidant effect of vitamin C can also manifest when it interacts with iron, reducing Fe^3+^ to Fe^2+^, or with copper, reducing Cu^2+^ to Cu^+^ [28,29]. The supplementation of vitamin C may result in a reduced normal biological response to free radicals and create an environment that is more susceptible to oxidation, potentially leading to mild oxidative stress due to its pro-oxidative properties [27]. Similarly, alpha-tocopherol, is another well-known potent antioxidant that can act as a pro-oxidant in high concentrations. This occurs due to its reaction to reactive oxygen species (ROS), where it remains in its reactive form without the availability of ascorbic acid [30,31].

Similarly, the same effect has been observed in another well-known category of antioxidants—flavonoids. Even flavonoids have been reported to act as pro-oxidants in systems containing transition metals. Flavonoids, such as quercetin and kaempferol, induce DNA damage and lipid peroxidation in the presence of transition metals [32]. Flavonoids can potentially act as pro-oxidants through several mechanisms, including direct interaction with oxygen via the Fl-O^●^ radical [33], inhibition of mitochondrial respiration, causing a substrate-independent cyanide-insensitive respiratory burst in isolated mitochondria, associated with the production of ROS [34], and oxidation by peroxidases, resulting in the formation of intracellular phenoxyl radicals by myeloperoxidase [35]. Finally, flavonoids can act as pro-oxidants by oxidizing low-molecular antioxidants [36].

A similar situation was observed for other phenolics in general. Phenolics can also display pro-oxidant effects, especially in systems containing redox-active metals. The presence of iron or copper catalyzes their redox cycling and may lead to the formation of phenolic radicals, which damage lipids and DNA [37,38].

The main goal of this paper was to investigate the simultaneous determination of antioxidant/Fe^3+^-reducing activity, producing pro-oxidant Fe^2+^ ions in randomly selected compounds and plant extracts on a microplate. The objective was to observe significant differences among the tested samples and initiate a systematic, rapid evaluation for further research.

## 2. Results and Discussion

In this study, we assessed the antioxidant activity of 30 randomly selected compounds and 18 plant extracts using the DPPH method, as well as their ability to reduce Fe^3+^ ions using the FRAP method, with TROLOX as a standard. The pro-oxidant antioxidant balance index (PABI) was calculated from the ratio FRAP_50_/DPPH_50_ according to Equation (4). The results for the tested compounds, along with their CAS numbers, DPPH_50_ and FRAP_50_ values, correlation coefficients, and the targeted PABI, are presented in Table 1. These compounds were randomly selected from our ongoing parallel studies. Several compounds were excluded from the dataset shown in Table 1 because their values were outside the testing interval.

The obtained results clearly indicate that the majority of the tested compounds exhibited both DPPH^●^ scavenging ability and Fe^3+^ reducing activity, thereby producing pro-oxidative Fe^2+^ ions. However, certain compounds such as rutin, β-carotene, and protocatechuic acid demonstrated antioxidant activity according to the DPPH method, yet their FRAP_50_ values exceeded the testing interval (1–4096 μM), suggesting lower pro-oxidant activity in comparison to their antioxidant activity. Based on the perspective of antioxidant activity determined by the DPPH method, the most potent compounds were the well-known flavonoids quercetin, morin, baicalein, 7,8-dihydroxyflavone, (−)-epicatechin, and polyphenolic acid-gallic acid, which exhibited higher antioxidant activity than the standard TROLOX. In the second category, compounds with DPPH_50_ values falling within the range of 115–500 μM were less active than the standard. This group included compounds such as rutin, hesperidin, (+)-catechin, l-ascorbic acid, tannic acid, caffeic acid, protocatechuic acid, and avenanthramide A and C.

When comparing antioxidant activity and the production of pro-oxidant Fe^2+^ ions using the PABI parameter, we can divide tested compounds into three categories: compounds with dominant pro-oxidant activity (PABI < 1), compounds with roughly balanced pro-oxidant/antioxidant activity (PABI from 1 to 2), and compounds with dominant antioxidant effect (PABI > 2). Compounds with PABI values less than 1 were considered pro-oxidants. These compounds included quercetin, tannic acid, and silibinin. On the other hand, compounds with PABI values within the interval of 1–3, representing a balanced pro-oxidant/antioxidant profile, included the standard TROLOX, morin, L-ascorbic acid, crocin, an anthraquinone purpurin, caffeic acid, and avenanthramide B. Remarkably, compounds with significantly high PABI values (over 5) included gallic acid (PABI = 8.06), and most notably, both tested catechins/(−)-epicatechin PABI = 12.4, (+)-catechin PABI = 23.25). Overall, both antioxidant and pro-oxidant activities varied throughout the entire testing concentration range (1–4096 μM), with PABI values spanning the interval of 0.41–23.25. These findings provide insight into the diversity of PABI values within the randomly selected compound collection, demonstrating a variation of over 50 times in this study. It is important to note that this ratio could change with an expansion of the tested compound collection. From a structural perspective, our findings support the thesis presented in the Introduction section [17], which suggested that a substantial reduction in ferric ions occurs under acidic conditions, particularly with flavonols and flavanols containing the catechol moiety in the B ring. Pro-oxidant activity refers to the ability to interact with transition metals, forming coordination complexes. These chemical compounds consist of a central atom or ion, which is usually metallic and is called the coordination center, surrounded by bound molecules or ions known as ligands or complexing agents. The coordination of Fe ions can occur with biomolecules possessing free ionic pairs in relatively close proximity, such as the catechol in the B ring or the keto group of flavonols and flavanols with an OH group in the vicinal position (position 3) on the C ring or near position (position 5) on the A ring. This may explain the higher pro-oxidant activity of flavonols and flavanols compared to catechins, which lack the sp2 hybridized keto group in position 4 of the C ring.

Our study’s findings indicate that flavonoids, particularly quercetin and flavolignan silibinin, exhibit more pronounced pro-oxidant properties due to specific structural features. These include the presence of a C2–C3 double bond in the C ring, the catechol conformation of vicinal hydroxyl groups, or the hydroxyl group in combination with the keto group at position 4 [33]. This observation aligns with existing literature [39,40], including other sources that suggest quercetin’s pro-oxidant effect contributes to its potential anticancer efficacy [41]. There are some records on the pro-oxidant activity of silibinin [42]. Contrary to our findings, previous publications have highlighted the pro-oxidant activity of catechins, particularly in the context of their anticancer effects [43,44]. It is important to acknowledge that our results are solely derived from in vitro studies, and this could partially explain the discrepancy. The saturated C-ring of catechins, lacking double bonds, may mitigate their pro-oxidant potential. A similar situation might be observed in gallic acid [45].

The second part of the study focused on screening randomly selected plant extracts prepared using an extraction process provided in the Material and Methods section. The study encompassed medicinal plants (*n* = 18), which are currently being explored. The achieved results are presented in Table 2.

Based on the results, there are some differences among medical plant extracts, although they are not as pronounced as those seen with single compounds. These variations can be attributed to the “buffer” effect of the complex mixtures found in these extracts, which could consist of numerous compounds. The antioxidant activity of the strongest samples, such as oak, birch leaves, bark, green tea leaves, common wormwood leaves, and rhubarb roots, is well documented in the literature. In our experiments, these samples exhibited DPPH_50_ values under 2 mg of extract-dried matter per milliliter. While there is less information in the literature regarding the ability to reduce Fe^3+^ ions and produce pro-oxidant Fe^2+^ ions, it is particularly interesting to interpret the results based on PABI values. Surprisingly, most of the tested extract samples (oak, rapeseed, rhubarb, wormwood, green tea, licorice, common wormwood, thistle, chamomile, or turmeric) had PABI values under 1, indicating a predominant pro-oxidant effect over antioxidants. In contrast, extract samples prepared from silver birch, horse chestnut, old man’s beard, black elderberry, ginger, sage, and notably grape wine showed a dominant antioxidant activity over pro-oxidant. One of the most promising findings is related to sage, which exhibited the most potent antioxidant activity among the samples with a high PABI index.

In the current body of literature, only a single paper focuses on the potential pro-oxidant effects of polyphenols from sage (and rosemary) [46], and only a handful of papers address grape wine [32,47], despite the abundance of publications discussing its antioxidant effects. Compared to our results, only a limited number of publications have described the simultaneous determination of antioxidant and pro-oxidant activities, preferably in a single format.

## 3. Materials and Methods

### 3.1. Chemicals and Solvents

2,2-Diphenyl-1-picrylhydrazyl radical (DPPH^●^), 2,2-Diphenyl-1-picrylhydrazin (DPPH), 2,4,6-Tris(2-pyridyl)-s-triazine (TPTZ), FeCl_2_·4H_2_O, FeCl_3_·6H_2_O, TROLOX, quercetin, rutin, baicalein, morin, 7,8-dihydroxyflavone, (−)-epicatechin, (+)-catechin, l-ascorbic acid, tannic acid, crocin, -caroten, purpurin, silibinin, olivetol, gallic acid, caffeic acid, protocatechuic acid, avenanthramides A, B, and C, flavone, ergocalciferol, lipoic acid, biotin, thiamin, indole-3-carbinol, astaxanthin, uric acid, sodium acetate, ethanol, and acetic acid were purchased from Merck /Sigma/ (St. Louis, MO, USA).

### 3.2. Preparation of the Extract Samples

Randomly selected plant material from 18 plant species (refer to Table 2) was chosen based on their bioactivity in our current research. A total of 2 g of dried plant matter was disintegrated into small pieces (under 5 mm particle size). The plant matter was then extracted in screwed-up tubes with 20 mL of a 50% ethanol solution in the dark at room temperature for 24 h. Afterward, the extract was filtered and stored in Eppendorf tubes at 4 °C in the dark.

### 3.3. Principles of Microplate Methods

**Figure 1:**
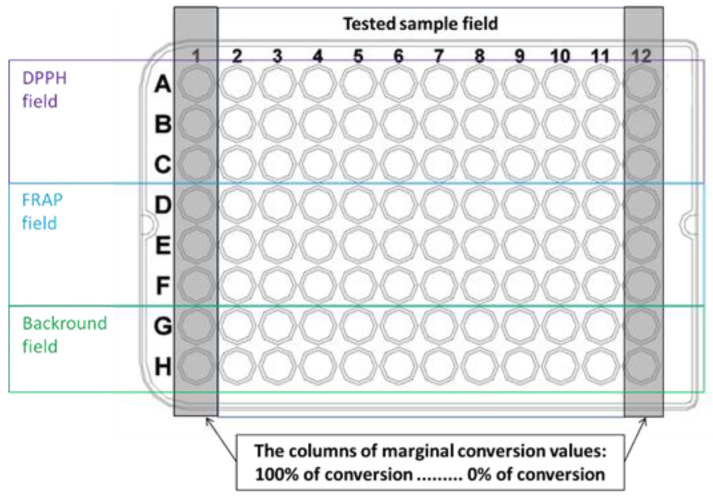
Microplate template. Organization of the microplate consisting of a tested sample field (wells A2–H12) and columns dedicated to standard, representing 100% conversion (wells A1–F1) and 0% conversion (wells A12–F12) for the assays.

The DPPH method was employed for measuring antioxidant activity, while the FRAP method was used to measure both antioxidant and pro-oxidant activity. Both methods were performed on microplates that were previously modified by our research team [4,5,6,7,8], ensuring equal concentrations of key reagents (DPPH, TPTZ, and FeCl3) at 0.4 mM. Briefly, a 0.4 mM DPPH^●^ radical solution was prepared in ethanol. For the FRAP assay, separate solutions A and B were prepared, with solution A containing 0.0187 g of TPTZ in 10 mL of ethanol and solution B containing 0.338 g of sodium acetate in 88.3 mL of water and 1.748 mL of acetic acid. These solutions were mixed freshly before each experiment. Additionally, 1.2 mM solutions of FeCl_2_·4H_2_O and FeCl_3_·6H_2_O were prepared freshly prior to the experiments. The microplate template used for the assays is presented in Figure 1.

### 3.4. Definition of the Conversion Intervals

In the first column (column 1), a 100% conversion standard for the DPPH method was applied using 2,2-diphenyl-1-picrylhydrazine (DPPH), and for the FRAP method, a solution of FeCl_2_·4H_2_O was used instead of the sample. In contrast, in the last column (column 12), 0% conversion standard for the DPPH method was applied using 2,2-Diphenyl-1-picrylhydrazyl radical (DPPH^●^), and for the FRAP method, a solution of FeCl_3_·6H_2_O was used instead of the sample.

### 3.5. Application of Tested Sample on Microplate

First, dilution of tested samples (compounds, extracts) in the microplate testing field was realized by both following dilution modes.

Mode 1, dilution to 1/2 concentration (100 μL of tested samples into column 2, 50 μL of ethanol into columns 3–10), transfer of 50 μL in the direction of columns 2–11 using one 8-channel micropipette. Starting from stock solution (for compounds) with a concentration of 8.192 mM, we achieve final concentrations in μM: 4096, 2048, 1024, 512, 256, 128, 64, 32, 16, and 8 μM. The extract samples were tested in 50% ethanol solutions as they were prepared.

Mode 2, dilution to 2/3 concentration (150 μL of tested samples into column 2, 50 μL of ethanol into columns 3–10), transfer of 100 μL in direction of columns 2–11 using one 8-channel micropipette, and removal of volume 100 μL from the last column. Starting from stock solution (for compounds) with optimal concentration determined by mode 1.

### 3.6. Application of Tested Sample on Microplate

Firstly, the dilution of tested samples (compounds and extracts) was realized on the microplate testing field by following both dilution modes. Mode 1, dilution to 1/2 concentration (100 µL of tested samples into column 2, 50 µL of ethanol into columns 3–10), transfer of 50 µL in the direction of columns 2–11 using one 8-channel micropipette. Starting from stock solution (for compounds) with a concentration of 8.192 mM, we achieve final concentrations in µM: 4 096, 2 048, 1 024, 512, 256, 128, 64, 32, 16, and 8 µM. The extract samples were tested as 50% ethanol solutions.

Mode 2, dilution to 2/3 concentration (150 µL of tested samples into column 2, 50 µL of ethanol into columns 3–10), transfer of 100 µL in the direction of columns 2–11 using one 8-channel micropipette, and removal of volume 100 µL from the last column. Starting from stock solution (for compounds) with optimal concentration determined by mode 1.

### 3.7. Preparation of the Microplate

Phase I—Application of the conversion standards:

- adding 50 μL of a 0.4-mM solution of 2,2-diphenyl-1-picrylhydrazine in ethanol into the wells A1–C1;
- adding 50 μL of a 1.2-mM water solution of FeCl_2_·4H_2_O into the wells D1–F1;
- adding 50 μL of a 0.4-mM solution of DPPH^●^ into the wells A12–C12;
- adding 50 μL of a 1.2-mM water solution of FeCl_3_·6H_2_O into the wells D12–F12.

Phase II—Application of FeCl_3_ solution and microplate filling:

- adding 50 μL of a 1.2-mM solution of FeCl_3_·6H_2_O into the field of three rows D2–F11,
- adding 150 μL of 50% (*v*/*v*) ethanol into the “background” wells in rows G and H (G2–H11).

### 3.8. Preparation of Starting Reagents

DPPH reagents were prepared as a 0.4-mM solution in ethanol freshly before the experiment. Similarly, FRAP reagent was prepared freshly before the experiment accordingly: 10 mL of a 6-mM TPTZ solution in ethanol and 90 mL of a sodium acetate-acetic buffer (0.338 g of sodium acetate dissolved in 88.5 mL of water, followed by the addition of 1.478 mL of acetic acid).

### 3.9. Starting and Adapting the Microplate before Measurement

The microplate was initialized by adding 150 μL of 0.4 mM DPPH reagent to rows A, B, and C, achieving a final DPPH concentration of 0.3 mM in the reaction mixture. Similarly, 100 μL of FRAP reagent was added to rows D, E, and F to achieve a final concentration of 0.3 mM for TPTZ and Fe^3+^ ions in the reaction mixture. Subsequently, 100 μL was removed from the whole microplate to achieve an optical density (OD) within the limit of the Lambert–Beer law.

### 3.10. Microplate Incubation and Measurement

The microplate was incubated for 10 min at room temperature, followed by measurements at 520 nm and 630 nm for DPPH and FRAP, respectively. The optical density (OD) data of the samples were adjusted by subtracting the background data and transformed into percentile values of conversion measurement using 0% and 100% conversion data. DPPH_50_ and FRAP_50_ parameters correspond to a concentration of a compound responsible for 50% conversion of either DPPH radical or Fe^3+^ to Fe^2+^ (expressed in μM). They were calculated from the following function plot: percentage of conversion = f(concentration). For extract samples, the concentration of dry matter weight of the extracted sample was used. The pro-oxidant antioxidant balance index /PABI/ was calculated according to Equation (4), using the micromolar expression for compounds and the weight of dried matter expression for extract samples.

Pro-oxidant Antioxidant Balance Index /PABI/ = FRAP_50_/DPPH_50_ (4)

### 3.11. Statistical Analysis of Data

Each experiment was repeated three times with eight replicates. Results were presented as the mean ± standard deviation (SD). The correlation coefficient was calculated using the Spearman method. A difference was considered statistically significant when * *p* < 0.1.

## 4. Conclusions

In summary, this study aimed at developing a rapid screening microplate method for simultaneous detection of the antioxidant and pro-oxidant activities of a diverse range of compounds and medical plant extracts. This study provides a preliminary overview, shedding light on the potential range of compounds spanning from those with strong antioxidant effects to those with pronounced pro-oxidant effects. Subsequent in-depth investigations are needed to build upon these initial insights.

Our upcoming research will focus on elucidating the structural characteristics that contribute to the prevalence of pro-oxidant effects. We plan to employ molecular mechanics and semi-empirical calculations using suitable software tools to delve into this aspect. Such analysis may be particularly beneficial for compounds commonly found in food, cosmetics, and pharmaceutical additives produced via plant suspension cultures. This will allow us to deepen our understanding of these compounds’ effects and implications.

## Figures and Tables

**Table 1:** Compounds tested, including CAS numbers, DPPH_50_, FRAP_50_ values, correlation coefficients (r^2^), and pro-oxidant antioxidant balance index (PABI).

STANDARD/ Compound	CAS	DPPH_50_ (μM)	r^2^	FRAP_50_ (μM)	r^2^	PABI
TROLOX	53188-07-1	115.01 ± 1.5	0.9895	171.14 ± 7.9	0.906	1.49
Quercetin	117-39-5	356.03 ± 1.8	0.954	156.26 ± 4.1	0.963	0.44
Rutin	153-18-4	400.81 ± 7.9	0.970	>4086	-	-
Baicalein	491-67-8	49.16 ± 1.4	0.958	186.19 ± 4.9	0.945	3.79
Morin	480-16-0	78.87 ± 2.2	0.915	176.85 ± 3.4	0.956	2.24
7,8-Dihydroxyflavone	38183-03-8	63.17 ± 1.4	0.939	253.91 ± 2.9	0.952	4.02
Hesperidin	520-26-3	523.24 ± 14.5	0.976	>4086	-	-
Diosmin	520-27-4	>4086	-	>4086	-	-
Apigenin-7-glucoside	578-74-5	>4086	-	>4086	-	-
(−)-Epicatechin	490-46-0	22.49 ± 0.5	0.950	279 ± 0.9	0.997	12.40
(+)-Catechin	154-23-4	192.63 ± 5.5	0.997	4479.36 ± 10.8	0.944	23.25
l-Ascorbic acid	50-81-7	164.06 ± 9.9	0.938	337.2 ± 0.8	0.938	2.06
Tannic acid	1401-55-4	111.82 ± 2.3	0.982	49.51 ± 0.5	0.974	0.44
Crocin	42553-65-1	771.1 ± 9.4	0.975	884.32 ± 17.2	0.960	1.15
β-Carotene	7235-40-7	2086 ± 29.5	0.971	>4086	-	-
Purpurin	81-54-9	941.3 ± 28.6	0.993	2504.61 ± 30.8	0.955	2.66
Silibinin	22888-70-6	3336.48 ± 107.1	0.974	1365.1 ± 22.7	0.929	0.41
Olivetol	500-66-3	>4086	0.939	>4086	-	-
Gallic acid	149-91-7	50.72 ± 0.8	0.931	408.97 ± 10.07	0.978	8.06
Caffeic acid	331-39-5	177.25 ± 8.8	0.937	500.39 ± 8.6	0.969	2.82
Protocatechuic acid	99-50-3	166.31 ± 8.1	0.982	>4086	-	-
Avenanthramide A	108605-70-5	448.6 ± 11.4	0.968	1730.67 ± 16.8	0.965	3.86
Avenanthramide B	108605-69-2	1566.2 ± 60.5	0.863	2778.45 ± 43.3	0.946	1.77
Avenanthramide C	116764-15-9	431.14 ± 20.6	0.989	1468.64 ± 14.3	0.971	3.41

**Table 2:** Randomly selected medical plant extracts, including DPPH_50_, FRAP_50_ values (expressed in mg of extract dried matter per milliliter), correlation coefficients (r^2^), and pro-oxidant antioxidant balance index (PABI).

Plant Species, Latin Name and Bot. Classifier	Plant Part	DPPH_50_ (mg dm/mL)	r^2^	FRAP_50_ (mg dm/mL)	r^2^	PABI
Sessile oak, *Quercus petraea*, (Matt.) Liebl.	leaves	2.54 ± 0.2	0.949	1.81 ± 0.22	0.980	0.71
Silver birch, *Betula pendula*, Roth.	leaves	1.6 ± 0.2	0.998	2.37 ± 0.3	0.986	1.48
Horse chestnut, *Aesculus hippocastanum*, L.	leaves	1.82 ± 0.1	0.979	9.09 ± 0.04	0.934	4.98
Old man’s beard, *Clematis vitalba*, L.	bark	7.72 ± 0.3	0.913	32.33 ± 0.2	0.944	4.18
Rapeseed, *Brassica napus*, L.	grains	145.15 ± 0.1	0.962	11.64 ± 0.3	0.961	0.08
Rhubarb, *Rheum rhabarbarum*, L.	root	1.39 ± 0.2	0.962	0.58 ± 0.1	0.933	0.42
Pedunculate oak, *Quercus robur*, L.	bark	0.5 ± 0.1	0.973	0.3 ± 0.1	0.939	0.61
Black elderberry, *Sambuicus nigra*, L.	flower	1.09 ± 0.1	0.968	1.61 ± 0.2	0.966	1.47
Woundwort, *Prunella vulgaris*, L.	flower	1.57 ± 0.1	0.965	1.27 ± 0. 1	0.962	0.81
Green tea, *Camelia sinensis*, (L.) Kuntze.	flower	1.93 ± 0.1	0.923	0.21 ± 0.1	0.993	0.11
Liquorice, *Glycyrrhiza glabra*, L.	root	5.62 ± 0.2	0.955	3.41 ± 0.2	0.967	0.61
Common wormwood, *Artemisia absinthium*, L.	leaves	1.73 ± 0.1	0.984	1.25 ± 0.1	0.963	0.73
Thistle, *Silybum marianum*, (L.) Gaertn.	grain	7.22 ± 0.4	0.991	1.61 ± 0.1	0.970	0.22
Chamomile, *Matricaria chamomilla*, L.	flower	5.06 ± 0.3	0.984	5.03 ± 0.2	0.981	0.99
Ginger, *Zingiber officinale*, Roscoe.	root	4.62 ± 0.2	0.994	5.43 ± 0.3	0.991	1.18
Turmeric, *Curcuma longa*, L.	root	17.52 ± 0.5	0.988	11.44 ± 0.4	0.982	0.65
Sage, *Salvia officinalis*, L.	leaves	0.27 ± 0.1	0.975	1.73 ± 0.1	0.959	6.27
Grape wine, *Vitis vinifera*, L.	frost dried grapes	3.52 ± 0.1	0.976	40.63 ± 1.8	0.961	11.53

## Data Availability

The data presented in this study are available on request from the corresponding author.
